# Alterations in Energy Metabolism, Mitochondrial Function and Redox Homeostasis in GK Diabetic Rat Tissues Treated with Aspirin

**DOI:** 10.3390/life12010104

**Published:** 2022-01-12

**Authors:** Annie John, Layla Amiri, Jasmin Shafarin, Saeed Tariq, Ernest Adeghate, Frank Christopher Howarth, Haider Raza

**Affiliations:** 1 Department of Biochemistry and Molecular Biology, College of Medicine and Health Sciences, UAE University, Al Ain P.O. Box 17666, United Arab Emirates; anniej@uaeu.ac.ae (A.J.); liamiri@dha.gov.ae (L.A.); jsalam@sharjah.ac.ae (J.S.); 2 Pathology Department, Latifa Women and Children’s Hospital, Dubai P.O. Box 9115, United Arab Emirates; 3 Sharjah Institute for Medical Research, University of Sharjah, Sharjah P.O. Box 27272, United Arab Emirates; 4 Department of Anatomy, College of Medicine and Health Sciences, UAE University, Al Ain P.O. Box 17666, United Arab Emirates; stariq@uaeu.ac.ae (S.T.); eadeghate@uaeu.ac.ae (E.A.); 5 Department of Physiology, College of Medicine and Health Sciences, UAE University, Al Ain P.O. Box 17666, United Arab Emirates; chris.howarth@uaeu.ac.ae

**Keywords:** GK rat, diabetes, redox metabolism, mitochondrial bioenergetics, aspirin, CYP 450s

## Abstract

Our recent studies have demonstrated that aspirin treatment prevents inflammatory and oxidative stress-induced alterations in mitochondrial function, improves glucose tolerance and pancreatic endocrine function and preserves tissue-specific glutathione (GSH)-dependent redox homeostasis in Goto-Kakizaki (GK) diabetic rats. In the current study, we have investigated the mechanism of action of aspirin in maintaining mitochondrial bioenergetics and redox metabolism in the liver and kidneys of GK rats. Aspirin reduced the production of reactive oxygen species (ROS) and oxidative stress-induced changes in GSH metabolism. Aspirin treatment also improved mitochondrial respiratory function and energy metabolism, in addition to regulating the expression of cell signaling proteins that were altered in diabetic animals. Ultrastructural electron microscopy studies revealed decreased accumulation of glycogen in the liver of aspirin-treated diabetic rats. Hypertrophic podocytes with irregular fusion of foot processes in the renal glomerulus and detached microvilli, condensed nuclei and degenerated mitochondria observed in the proximal convoluted tubules of GK rats were partially restored by aspirin. These results provide additional evidence to support our previous observation of moderation of diabetic complications by aspirin treatment in GK rats and may have implications for cautious use of aspirin in the therapeutic management of diabetes.

## 1. Introduction

Despite decades of extensive research and medical breakthroughs, there is still no therapeutic silver bullet to prevent the progression of diabetes and its complications, which are increasing alarmingly worldwide. As per the International Diabetes Federation (IDF), global estimates of diabetes prevalence are expected to increase to 693 million by 2045 [1]. Type 2 diabetes mellitus (T2D) is the most common endocrine metabolic disorder, characterized by impaired insulin response and insulin resistance. Metabolic reprogramming and adaptation in energy metabolism, play an important role in the progression of diabetes. The Goto-Kakizaki (GK) rat, a non-obese and spontaneous (genetic) model of T2D, exhibits alterations in β-cell response, impaired glucose-induced insulin secretion, glucose intolerance, peripheral insulin resistance and chronic inflammation [2,3,4]. These animals are widely used to study T2D and its complications [5,6]. Glucolipotoxicity in diabetes is associated with increased inflammation, oxidative stress and mitochondrial dysfunction, which contribute significantly in the development of metabolic complications/syndrome [7,8,9,10,11]. Since chronic inflammation has been implicated in the pathophysiology of T2D, targeting inflammation improved insulin sensitivity and β-cell function in patients with T2D or other metabolic disorders [7]. Our previous studies using both type 1 and type 2 diabetic models have shown tissue-specific alterations in redox metabolism, oxidative stress and mitochondrial functions [8,9,10,11].

Non-steroidal anti-inflammatory drugs (NSAIDs), including aspirin, known mainly for their anti-inflammatory, antipyretic and analgesic effects, have shown beneficial effects in diabetes prevention/treatment [12]. Aspirin is a multifunctional drug, with different mechanisms, including inhibition of platelet function via the acetylation of cyclooxygenase-1 (COX-1) and inhibition of prostaglandin production via the acetylation of COX-2, expressed by cytokines and other inflammatory stimuli [13]. More recently, low dose aspirin has been suggested as an important pharmacological strategy to prevent the ‘cytokine storm’ commonly seen in COVID-19 patients [14]. However, there are conflicting reports that NSAIDs cause nephrotoxicity, gastritis and bleeding [13,15,16]. Though the mechanism of toxicity is not fully understood, it is proposed to be a combination of inhibition of COX-1 and/or uncoupling of mitochondrial oxidative phosphorylation [17]. Furthermore, studies have shown gastric toxicity only when aspirin was administered orally in animal models [18,19]. No gastric damage or mitochondrial uncoupling was observed after intraperitoneal administration of aspirin in rats [17,20].

Our study in aspirin-treated GK rats showed a marked reduction in serum prostaglandin E2 (PGE2) levels and an increase in nitric oxide (NO) production, which may have stimulated glucose tolerance and increased insulin response [21]. Besides inflammation, chronic glucolipotoxicity is known to cause nephropathy and non-alcoholic fatty liver disease (NAFLD) in diabetes [22]. Our recent study in the pancreas and heart of aspirin-treated GK rats [23] demonstrated improved mitochondrial function, which in turn facilitated better energy utilization in these tissues. We used the same cohort of animals to further elucidate the alterations in redox homeostasis and mitochondrial bioenergetics in the liver and kidney tissues and investigate the effect of aspirin on these alterations. Our study demonstrates that aspirin treatment exerted beneficial effects on GK diabetic rats via modulation of redox homeostasis, mitochondrial function and energy metabolism. This is the first in vivo study showing the direct beneficial impact of aspirin on mitochondrial bioenergetics and redox homeostasis in liver and kidney affected by the metabolic complications of diabetes.

## 2. Materials and Methods

### 2.1. Chemicals

Aspirin (acetylsalicylic acid, ASA), lucigenin, reduced glutathione (GSH), oxidized glutathione (GSSG), 5,5′-dithio-bis (2-nitrobenzoic acid), 1-chloro 2,4-dinitrobenzene (CDNB), cumene hydroperoxide, dimethyl nitrosamine (DMNA), erythromycin, glutathione reductase, NADH, NADPH, coenzyme Q2, antimycin A, dodecyl maltoside, sodium succinate, cytochrome c, lucigenin, an ATP bioluminescent somatic cell assay kit (# FLASC) and hexokinase colorimetric assay kits (# MAK091) were purchased from Sigma-Aldrich Fine Chemicals (St. Louis, MO, USA). 2′, 7′-Dichlorofluorescein diacetate (DCFDA) was procured from Molecular Probes (Eugene, OR, USA). Polyclonal antibodies against protein kinase B (Akt) (# 4691), phosphorylated protein kinase B (p-Akt) (# 4060), phosphorylated c-jun N-terminal kinase (p-JNK) (# 9255) and glyceraldehyde 3-phosphate dehydrogenase (GAPDH) (# 2118) were purchased from Cell Signaling Technology, Inc. (Danvers, MA, USA). Antibodies against c-jun N-terminal kinase (JNK) (# sc-7345) were purchased from Santa Cruz Biotechnology Inc. (Santa Cruz, CA, USA) and against heme oxygenase-1 (HO-1) (# ab13248) and glucose transporter-4 (Glut-4) (# ab654) from Abcam (Cambridge, UK). Kits for superoxide dismutase (SOD) (#ab65354) and glutamate dehydrogenase (GDH) (# ab102527) were procured from Abcam (Cambridge, UK) and lipid peroxidation (LPO) kits (# Bioxytech^®^ LPO-586TM) from Oxis Int. Inc. (Portland, OR, USA). Reagents for SDS-PAGE and Western blot analyses were purchased from Gibco BRL (Grand Island, NY, USA) and Bio Rad Laboratories (Richmond, CA, USA).

### 2.2. Animals

Five-week-old male GK rats (*n* = 10, each weighing around 100–120 g) were purchased from Taconic (Germantown, NY, USA). Male Wistar rats (*n* = 10) of similar age and weight were obtained from the Animal House Facility of College of Medicine and Health Sciences, United Arab Emirates University (U.A.E) and were used as non-diabetic controls. All animals were maintained under a standard 12-h light/dark cycle with free access to food and water ad libitum. Approval for this project (No. A1-13 dated 24 September 2013) was obtained from the Animal Research Ethics Committee, College of Medicine and Health Sciences, UAE University, UAE Research guidelines as stipulated by the NIH, USA, for safe practice for animals were followed while handling these animals. Blood glucose levels and weights of the animals were systematically monitored weekly from the start of ASA administration until the end of the experiment.

### 2.3. Experimental Design

At 4 months of age, the GK rats (avg. body wt. 319 g) showed impaired glucose tolerance with mild hyperglycemia and dyslipidemia. This was confirmed in our previous study [21]. The animals were divided into four groups: control, control + ASA, GK and GK + ASA. Each group consisted of five animals. The ASA groups of GK and Wistar control rats were injected intraperitoneally with 100 mg aspirin (ASA)/kg body weight/day for 5 weeks (100 mg/mL aspirin was prepared in normal saline and sodium hydroxide pellets were slowly added to completely dissolve the aspirin crystals and to neutralize the pH). Depending on their weights, animals in the aspirin (ASA) treatment groups were injected with 250–350 µL of 100 mg/mL aspirin solution. Similarly, control animals in both the groups were injected with an equivalent amount (250–350 µL) of normal saline, depending on their weights. The experimental dose and time point were selected based on previous published reports, as well as our study, using diabetic models including GK rats [21,23,24,25,26]. Minimal gastric or renal toxicity were seen in experimental animal models at this dose and using this route of administration [15,17,18,19,20]. The body weights and random blood glucose levels of animals in all the four groups, were monitored for 5 weeks from the day of injection until the end of the experiment.

The experiment was terminated at the end of five weeks and the animals were sacrificed via decapitation. Liver and kidney from the animals were quickly excised and stored at −80 °C until further analysis. Samples of fresh liver and kidney tissues were fixed immediately in Karnovsky’s fixative, pH 7.2, for ultrastructural studies. Portions of the tissues were homogenized (25% *w*/*v*) in H-medium (70 mM sucrose, 220 mM mannitol, 2.5 mM HEPES, 2 mM EDTA and 0.1 mM phenylmethylsulphonyl fluoride, pH 7.4) and used for isolating mitochondria, cytosol and microsomal fractions via differential centrifugation, and the purity of the mitochondrial preparation was checked routinely as previously described [10]. The protein concentration was measured using Bio-Rad reagent as previously described [9,21].

### 2.4. Measurement of Reactive Oxygen Species (ROS), NADPH Oxidase (NOX) Activity, Lipid Peroxidation (LPO) and Superoxide Dismutase (SOD) Activity

Determination of total peroxides, generated from free radicals, which accounts for ROS production in the liver and kidney of the control and diabetic animals with/without aspirin, was measured using the DCFDA fluorescence method as previously described [8,9,10,11]. To complement the measurement of ROS production, NADPH oxidase (NOX) activity was measured using a lucigenin-enhanced chemiluminescence assay and the chemiluminescence signals read using a TD-20/20 luminometer (Turner Designs, Sunnyvale, CA, USA). Lipid peroxidation was measured using an LPO-586™ assay kit (# Bioxytech^®^ LPO-586TM) as per the manufacturer’s recommendations, and the concentration of malonedialdehyde (MDA) was calculated from the standard curve using MDA as standard as previously described [27]. To further assess the antioxidant status in the liver and kidney of the control and diabetic animals with/without aspirin, superoxide dismutase (SOD) activity was measured based on the percent conversion of nitroblue tetrazolium (NBT) to NBT-diformazan as per the vendor’s protocol (R & D Systems Inc, MN, USA). Appropriate positive controls were used as recommended by the vendor. The percent reduction in formazan formation was used as the measure of SOD activity.

### 2.5. Measurement of Glutathione (GSH)-Redox Metabolism

GSH, the body’s major antioxidant, plays a key role in protecting organs from oxidative insults. Thus, alterations in GSH-redox metabolism is a vital indicator of disturbances in antioxidant metabolism. GSH levels were measured via the enzymatic conversion of oxidized glutathione (GSSG) to reduced glutathione (GSH) using Ellman’s reagent. Glutathione-S-transferase (GST) activity, glutathione peroxidase (GSH-Px) activity and glutathione reductase activity using 1-chloro 2,4-dinitrobenzene (CDNB), cumene hydroperoxide and GSSG/NADPH as respective substrates were measured using standard protocols as previously described [8,10].

### 2.6. Activities of Energy Metabolizing Enzymes

Activity of hexokinase, the rate-limiting enzyme in the glycolytic pathway, was measured using a hexokinase colorimetric assay kit (Sigma-Aldrich, St. Louis, MO, USA) as per the vendor’s protocol. Briefly, it is a coupled enzyme assay, resulting in the production of NADH, which reduces a colorless probe, producing a colorimetric (450 nm) product proportional to the hexokinase activity.

Activity of glutamate dehydrogenase (GDH), a mitochondrial enzyme, was measured using a GDH kit (Abcam, Cambridge, UK) as per the manufacturer’s instructions. Briefly, mitochondria from the different tissues were treated with a reaction buffer, containing glutamate as a specific substrate and the NADH generated was determined spectrophotometrically using the UV-1800 spectrophotometer (Schimadzu Corporation, Kyoto, Japan) at 450 nm.

### 2.7. Measurement of Mitochondrial Respiratory Enzyme Complexes and Bioenergetics

Freshly isolated mitochondria (5 μg protein) from the aspirin-treated and untreated control and GK rat liver and kidneys were suspended in 20 mM KPi buffer, pH 7.4, in the presence of a detergent, dodecyl maltoside (0.2%). Complex I (NADH-ubiquinone oxidoreductase), complex II/III (succinate-cytochrome reductase) and complex IV (cytochrome c oxidase) activities were measured using the substrates, coenzyme Q2 and succinate and reduced cytochrome c, respectively, according to the methods of Birch-Machin and Turnbull [28] as previously described [9,10,11]. The ATP levels were measured using the ATP Bioluminescent cell assay kit (Sigma, St. Louis, MO, USA) as per the vendor’s protocol and luminescence was read using a TD-20/20 luminometer (Turner Designs, Sunnyvale, CA, USA).

### 2.8. Measurement of Cytochrome P450 (CYP 450)-Dependent 2E1 and 3A4 Enzymes

Cytochrome P450-dependent enzymes play an important role in detoxification and metabolizing various drugs and xenobiotics. Our previous studies have shown that these enzymes are affected in diabetes, causing oxidative stress and mitochondrial dysfunction [8,10,11]. CYP 2E1-dependent N-demethylase activity and CYP 3A4 activities were measured in the microsomal fraction of liver and kidney from aspirin-treated and untreated control animals using dimethyl nitrosamine and erythromycin as specific substrates respectively, using standard methods as previously described [8,10,11].

### 2.9. SDS-PAGE and Western Blot Analysis

Tissue homogenates (50 μg protein) from liver and kidney of aspirin-treated and untreated control rats were separated using 12% SDS-PAGE [29] and electrophoretically transferred onto nitrocellulose membrane via Western blotting [30]. The membranes were then immunoblotted with oxidative stress marker protein (HO-1) and cell signaling regulatory proteins (p-Akt, Akt, Glut-4, p-JNK and JNK) and developed using Pierce Western blot substrate as previously described [8,9,10,11]. GAPDH was used as the loading control. Densitometric analysis was performed using the Typhoon FLA 9500 system (GE Healthcare, Uppsala, Sweden) and expressed as relative ratios normalized to GAPDH or their respective total proteins, as appropriate.

### 2.10. Transmission Electron Microscopy (TEM)

Tissue samples from liver and kidney of ASA-treated and untreated control rats were fixed in Karnovsky’s fixative, pH 7.2, for 24 h at 4 °C. The tissue specimens were then washed three times with 0.1 M phosphate buffer, pH 7.2, for 15 min each, followed by fixation in 1% aqueous solution of osmium tetroxide for one hour. The sections were then dehydrated in increasing concentrations (50–100%) of ethanol and finally dipped in propylene oxide. The tissue specimens were then infiltrated with a mixture of propylene oxide and agar epoxy resin mixture in different ratios and finally embedded in a mold with pure resin, in an embedding oven at 65 °C for 24 h for polymerization. The blocks were then trimmed and semi-thin (1.5 μm) and ultrathin (95 nm) sections were cut using a Leica EM UC7 (Vienna, Austria) ultra-microtome. The ultrathin sections were contrasted with Uranyl acetate followed by lead acetate, dried on filter paper and then observed under a FEI Tecnai G2 Spirit (Netherlands) transmission electron microscope. Images were captured at different magnifications as previously described [31]. The number of mitochondria and the mitochondrial area were quantified using ImageJ 1.53e software (National Institute of Health). At least 10 electron micrographs/group for each of the tissues were analyzed and the mean calculated and histograms plotted.

### 2.11. Statistical Analysis

Values shown are expressed as mean ± SD of three individual experiments. The statistical significance of the data was assessed using SPSS software (version 23) by means of analysis of variance followed by least significant difference (LSD) post-hoc analysis, and statistical significance was set at *p* < 0.05.

## 3. Results

### 3.1. Body Weights and Blood Glucose Levels after Treatment with ASA

**Figure 1:**
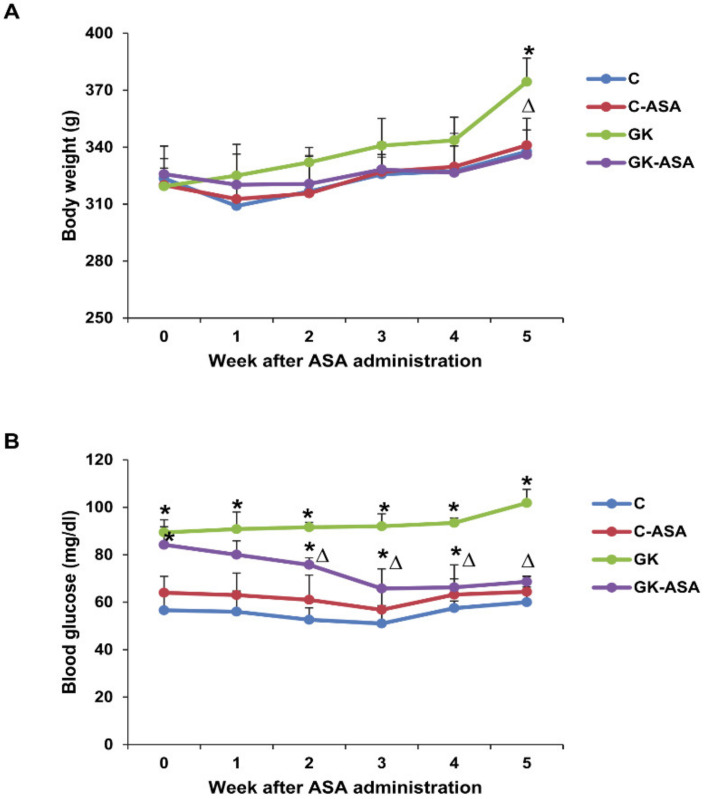
Body weights and blood glucose levels of control Wistar (C) and GK rats with/without aspirin (ASA) treatment (*n* = 5). Body weights (**A**) and blood glucose levels (**B**) were monitored regularly for 5 weeks from the day of aspirin injection. Results are expressed as mean ± SD from three independent experiments and asterisks indicate significant difference, which was fixed as *p* < 0.05 (* indicates *p* < 0.05 compared with control and Δ indicates *p* < 0.05 compared with GK rats).

Body weights and random blood glucose levels were monitored weekly from the start of aspirin treatment until the end of the experiment. All four groups showed a linear increase in body weights at the end of the experiment (Figure 1A). By the end of 5 weeks, the GK rats showed a significant increase in body weight compared with the control animals. Similarly, blood glucose levels had progressed significantly in the GK animals by the end of 5 weeks (Figure 1B). Aspirin treatment significantly decreased the glucose levels, bringing them close to the control values.

### 3.2. Effect of Aspirin on Oxidative Stress

**Figure 2:**
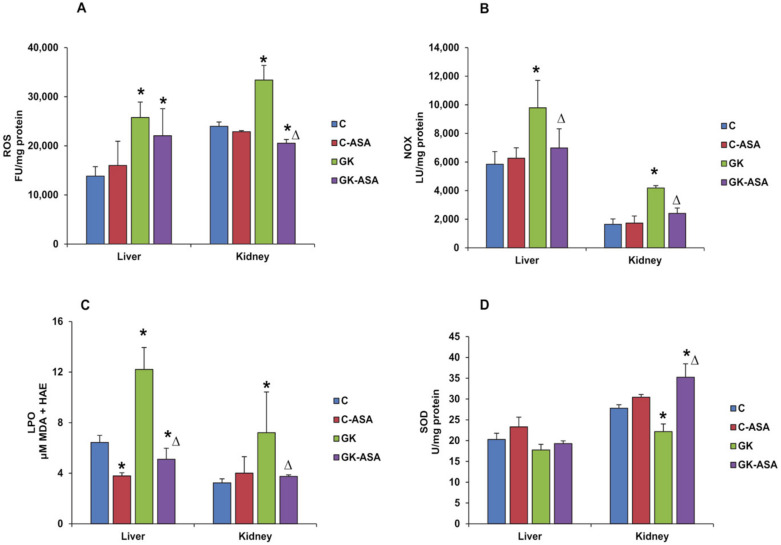
Measurement of ROS, NOX, lipid peroxidation (LPO) and SOD activity in control Wistar (C) and GK rats treated with/without aspirin (*n* = 5). Production of ROS (**A**) in the liver and kidney was measured using the DCFDA fluorescence method, and NOX activity (**B**) was measured using a lucigenin-enhanced chemiluminescence assay and measured using a TD-20/20 luminometer (Turner Designs, Sunnyvale, CA). Lipid peroxidation (**C**) was measured using an LPO-586™ assay kit as per the manufacturer’s recommendations, and the concentration of malonedialdehyde (MDA) was calculated from the standard curve using MDA as the standard. SOD activity (**D**) was measured based on the percent conversion of NBT to NBT-diformazan as per the vendor’s protocol. Results are expressed as mean ± SD from three independent experiments and asterisks indicate significant difference, which was fixed as *p* < 0.05 (* indicates *p* < 0.05 compared with control and Δ indicates *p* < 0.05 compared with GK rats).

Derivatives of free radicals, such as hydrogen peroxide, which accounts for the reactive oxygen species (ROS) generated by metabolic pathways, play crucial physiological roles in cell signaling and functioning. However, increase above physiological levels results in oxidative stress, causing complications in metabolic disorders including diabetes. We observed a significant increase (20–40%) in ROS production in GK diabetic rat liver and kidney (Figure 2A). Aspirin treatment reduced ROS production significantly in the kidney of GK animals, but in the liver, ROS production remained higher than in the control animals. No significant change in ROS production was observed in the liver and kidney of control animals treated with aspirin. We further checked the NADPH oxidase (NOX) activity in the tissues of control and GK animals treated with/without aspirin. A similar increase (almost 2–fold) was seen in the liver and kidney of GK rats (Figure 2B). Aspirin treatment significantly reduced the activity in both the tissues of the GK rats. Control animals, however, showed no significant changes after aspirin treatment. Similarly, lipid peroxidation, as measured by the end products, MDA and 4-hydroxyalkenals (HAE), was also significantly (40–80%) increased in both the liver and kidney of GK diabetic rats (Figure 2C). Aspirin treatment reduced the lipid peroxidation more drastically in the liver compared with the kidney. This effect was also seen in the liver of control rats treated with aspirin. The kidney of these control animals, however, showed a mild increase in lipid peroxidation. SOD enzyme activity, on the other hand, was decreased (~13%) in the liver but significantly (~20%) in the kidney. Aspirin treatment increased SOD activity in the kidney but not in the liver (Figure 2D). This indicates increased oxidative stress response in the kidney compared with the liver. However, a moderate increase in SOD activity was also observed in the liver and kidney of control rats treated with aspirin.

### 3.3. Effect of Aspirin on GSH-Redox Metabolism

**Figure 3:**
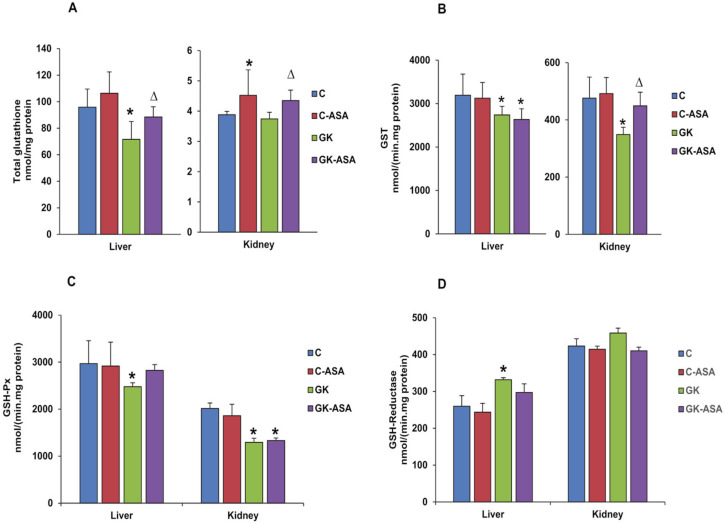
Effect of aspirin (ASA) on GSH metabolism in control Wistar (C) and GK rats treated with/without aspirin (*n* = 5). Total glutathione levels (**A**) in the liver and kidney were measured via the enzymatic conversion of oxidized glutathione to reduced glutathione using Ellman’s reagent. Glutathione-S-transferase (GST) activity (**B**), glutathione peroxidase (GSH-Px) (**C**) activity and glutathione reductase (**D**) activity were measured using CDNB, cumene hydroperoxide and GSSG/NADPH as respective substrates using standard protocols as described in Materials and Methods. Results are expressed as mean ± SD from three independent experiments and asterisks indicate significant difference, which was fixed as *p* < 0.05 (* indicates *p* < 0.05 compared with control and Δ indicates *p* < 0.05 compared with GK rats).

Oxidative stress due to high ROS production contributes to redox imbalance, which could be due to glutathione depletion. Our study showed that the total glutathione (GSH + GSSG) levels decreased significantly (~30%) in the liver compared with the kidney of GK diabetic rats. Aspirin treatment resulted in the recovery of depleted GSH in both the tissues of GK rats (Figure 3A). Aspirin treatment significantly increased the total glutathione levels in the liver and kidney of control rats. Activity of GST, the GSH-conjugating and detoxifying enzyme, was however reduced in both the liver and kidney of GK rats. Aspirin treatment improved the activity in the kidney but not in the liver (Figure 3B). The kidney of control rats also showed a mild increase in activity compared with the liver. Similarly, GSH-Px enzyme activity was also significantly inhibited in the liver (20%) and kidney (40%) of GK rats. However, a slight recovery was observed only in the liver after aspirin treatment (Figure 3C). These results suggest increased oxidative stress responses in the kidney compared with the liver. Control rats, on the other hand, showed no significant change after treatment with aspirin. In contrast, GSH-reductase enzyme activity increased significantly in the liver (30%) of GK rats compared with the kidney (Figure 3D). These observations of decreased GSH-Px activity, in combination with no significant increase in GSH-reductase activity, clearly indicate that the kidneys are subject to enhanced oxidative stress and damage due to unstable GSH-redox homeostasis. The control animals showed no significant change after treatment with aspirin.

### 3.4. Effect of Aspirin on Energy Metabolism and Mitochondrial Function

**Figure 4:**
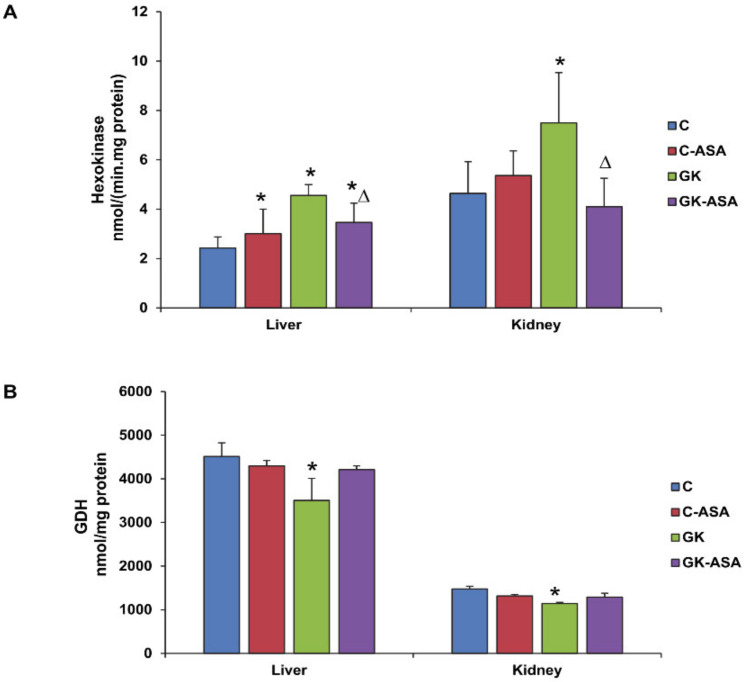
Effect of aspirin (ASA) on hexokinase and glutamate dehydrogenase activities in control Wistar (C) and GK rats treated with/without aspirin (*n* = 5). Hexokinase activity (**A**) in the liver and kidney was measured spectrophotometrically as NADH generated using a kit as per the manufacturer’s instructions. Glutamate dehydrogenase activity (**B**) was measured using a kit as a coupled enzyme assay as per the vendor’s protocol. Results are expressed as mean ± SD from three independent experiments and asterisks indicate significant difference, which was fixed as *p* < 0.05 (* indicates *p* < 0.05 compared with control and Δ indicates *p* < 0.05 compared with GK rats).

To study the alterations in glucose metabolism after aspirin treatment in GK rats, we checked the activity of the glycolytic enzyme, hexokinase, which was significantly increased (30–60%), both in the kidney and in the liver. Aspirin treatment reduced the activities significantly in both the tissues (Figure 4A). The enhanced hexokinase activity in tissues might suggest an increased adaptation to phosphorylate glucose in response to the mild hyperglycemia in GK rats. On the other hand, activity of a key Krebs’ cycle enzyme, glutamate dehydrogenase (GDH), was found to be reduced in both the liver and kidney of GK rats (Figure 4B). No significant change in activity was observed after aspirin treatment in both the tissues.

**Figure 5:**
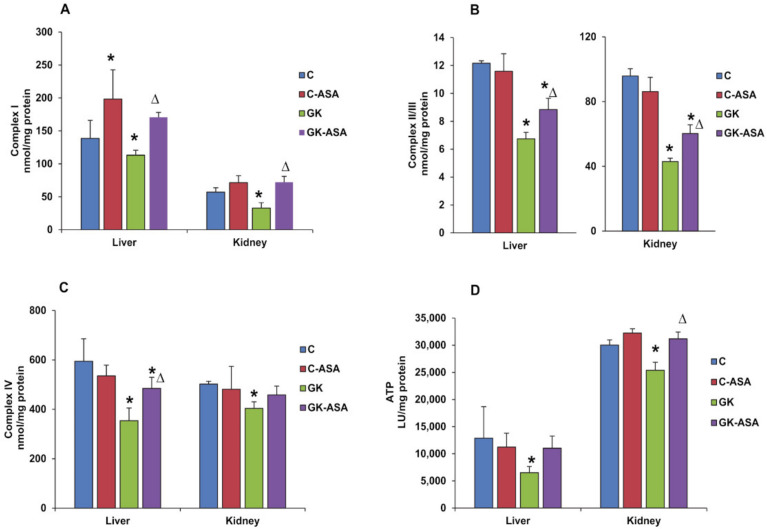
Effect of aspirin (ASA) on mitochondrial respiratory functions. Freshly isolated mitochondria (5 μg protein) from the aspirin treated/untreated control (C) and GK rat liver and kidneys were assayed for complex I (**A**), complex II/III (**B**) and complex IV (**C**) activities using the substrates, coenzyme Q2, succinate and reduced cytochrome c, respectively, as described in the Materials and Methods [28]. ATP levels (**D**) were measured using an ATP bioluminescent cell assay kit (Sigma, St. Louis, MO, USA) as per the vendor’s protocol. Results are expressed as mean ± SD from three independent experiments and asterisks indicate significant difference, which was fixed as *p* < 0.05 (* indicates *p* < 0.05 compared with control and Δ indicates *p* < 0.05 compared with GK rats).

To further investigate the precise role and mechanism of aspirin in maintaining the mitochondrial bioenergetics function, we studied the activities of the mitochondrial respiratory complexes, which showed a marked reduction in complex I, II/III and IV enzyme activities both in the liver and in the kidney of GK rats (Figure 5A–C). Aspirin treatment normalized complex I and II/III enzyme activities in the liver and kidney of GK rats. However, significant increase in complex IV enzyme activity after aspirin treatment was observed only in the liver of GK rats. Similarly, ATP production was also reduced in both the tissues of GK rats, which was recovered in the kidney after aspirin treatment (Figure 5D). These results suggest a compromised energy metabolism in GK diabetic rats and the beneficial effects of aspirin treatment.

### 3.5. Effect of Aspirin on CYP450 Enzyme Activities

**Figure 6:**
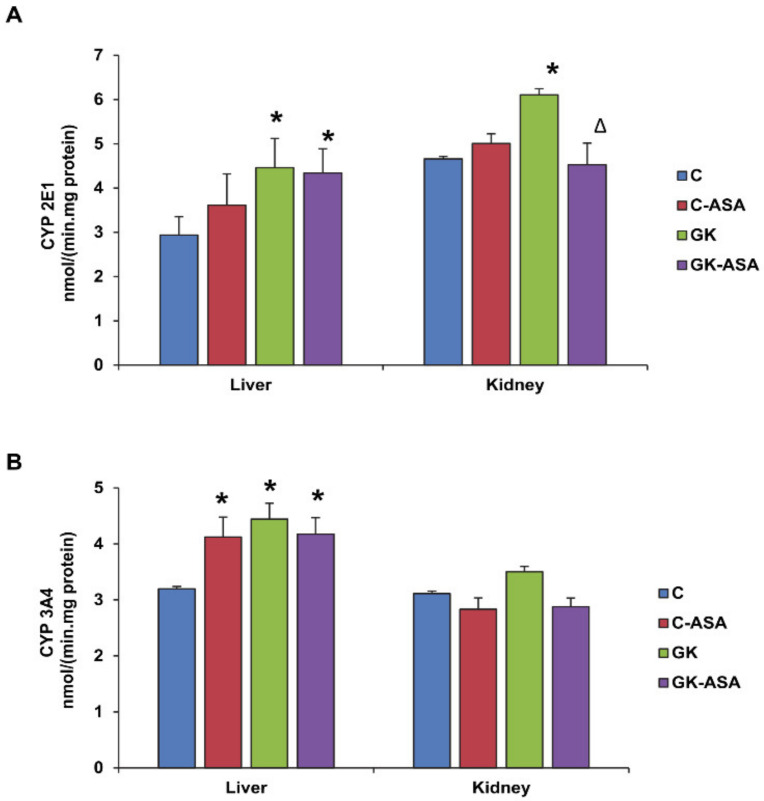
Effect of aspirin (ASA) on cytochrome P450 activities. Microsomal CYP 2E1 (**A**) and CYP 3A4 (**B**) activities were measured in control Wistar (C) and GK rats treated with/without aspirin (*n* = 5) using isoenzyme-specific substrates as described in Materials and Methods. Results are expressed as mean ± SD from three independent experiments and asterisks indicate significant difference, which was fixed as *p* < 0.05 (* indicates *p* < 0.05 compared with control and Δ indicates *p* < 0.05 compared with GK rats).

The phase I drug metabolizing cytochrome P450-dependent enzymes, which play an important role in detoxification, has been shown to be affected in diabetes, thus causing oxidative stress. Our present study showed enhanced activities of the CYP450 enzymes, CYP 2E1 (Figure 6A) and CYP 3A4 (Figure 6B) in GK diabetic compared with control rat liver and kidney suggesting increased metabolism of endogenous substrates (such as ROS, lipids and their metabolites) along with increased ability to metabolize xenobiotics and drugs as an adaptation mechanism in these animals. Both CYP 2E1 and CYP 3A4 activities remained significantly increased in the liver of GK rats and control rats after aspirin treatment. However, aspirin treatment resulted in a significant decrease in CYP 2E1 activity only in the kidney of GK rats. A mild increase in CYP 3A4 activity was observed in the kidney of GK rats, which normalized to control levels after aspirin treatment. This again confirms differential tissue-specific response of drug-metabolizing enzymes to the metabolism of endogenous and exogenous substrates.

### 3.6. Effect of Aspirin on Heme Oxygenase (HO-1) Expression

**Figure 7:**
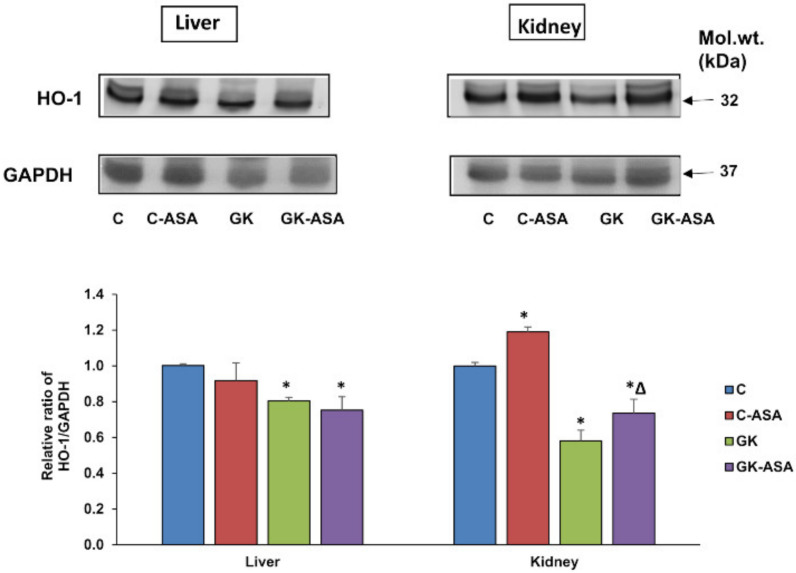
Effect of aspirin (ASA) on the expression of oxidative stress marker protein HO-1. SDS-PAGE separation of proteins from aspirin treated/untreated control and GK rat liver and kidney using 12% acrylamide gels and transfer onto nitrocellulose paper was performed using standard procedures as described in Materials and Methods. Immunoreactivity was measured using the specific antibody to HO-1. GAPDH was used as loading control. Results shown are representative blots from three independent experiments. The lower bands were used for the quantification, which was done using the Typhoon FLA 9500 system (GE Healthcare, Uppsala, Sweden). Histograms represent relative expression of the protein compared to the control. Asterisks indicate significant difference, which was fixed as *p* < 0.05 (* indicates *p* < 0.05 compared with control and Δ indicates *p* < 0.05 compared with GK rats and molecular weights of the respective proteins are shown in kilo Dalton (kDa). Original western blot figures are shown in Figure S1 and quantitation values shown in Table S1.

To check the effect of aspirin on antioxidative defense response in diabetes, we checked the expression of an oxidative stress responsive enzyme, HO-1 protein, which was significantly decreased in both the tissues of GK diabetic rats (kidney ~40% and liver ~20%) (Figure 7). Aspirin treatment enhanced the depleted enzyme expression only in the kidney of GK rats and control rats, suggesting modulation of altered redox responses in the kidney. However, no significant change was observed in the liver of GK or control rats after aspirin treatment.

### 3.7. Effect of Aspirin on Cell/Insulin Signaling Akt/Glut-4/JNK Markers

**Figure 8:**
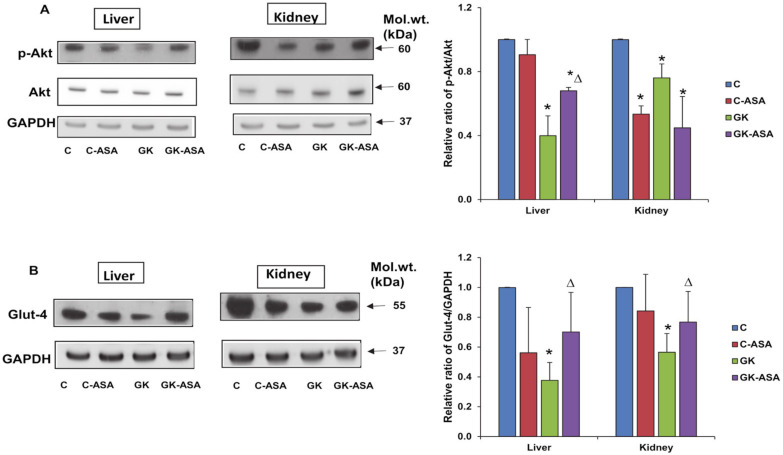
Effect of aspirin (ASA) on the expression of cell-signaling regulatory protein Akt (**A**) and the glucose transporter protein Glut-4 (**B**). SDS-PAGE separation of proteins from aspirin treated/untreated control and GK rat liver and kidney using 12% acrylamide gels and transfer onto nitrocellulose paper was performed using standard procedures as described in Materials and Methods. Immunoreactivity was measured using the specific antibody to Akt, p-Akt and Glut-4 proteins. GAPDH was used as loading control. Results shown are representative blots from three independent experiments. The lower bands (p-Akt) and Glut-4 immunoreactive bands were quantitated using the Typhoon FLA 9500 system (GE Healthcare, Uppsala, Sweden). Histograms represent the relative ratio of the phosphorylated protein compared to the total protein (for p-Akt) and GAPDH (for Glut-4). Asterisks indicate significant difference, which was fixed as *p* < 0.05 (* indicates *p* < 0.05 compared with control and Δ indicates *p* < 0.05 compared with GK rats). Molecular weights of the respective proteins are shown in kilo Dalton (kDa). Original western blot figures are shown in Figures S2 and S3, quantitation values shown in Tables S2 and S3.

**Figure 9:**
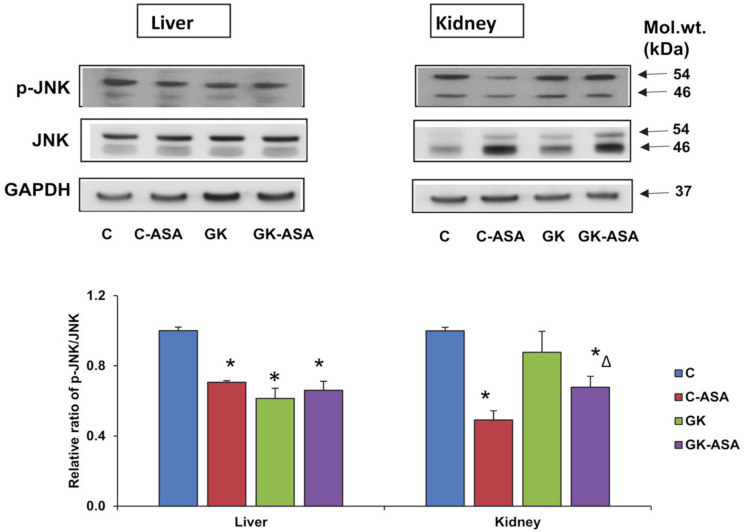
Effect of aspirin (ASA) on the expression of cell-signaling regulatory protein JNK. SDS-PAGE separation of proteins from aspirin treated/untreated control and GK rat liver and kidney using 12% acrylamide gels and transfer onto nitrocellulose paper was performed using standard procedures as described in Materials and Methods. Immunoreactivity was measured using the specific antibody to JNK and p-JNK. GAPDH was used as loading control. Results shown are representative blots from three independent experiments. The upper bands (p54) were used for the quantification, which was done using the Typhoon FLA 9500 system (GE Healthcare, Uppsala, Sweden). Histograms represent the relative ratio of the phosphorylated protein compared to the total protein. Asterisks indicate significant difference, which was fixed as *p* < 0.05 (* indicates *p* < 0.05 compared with control and Δ indicates *p* < 0.05 compared with GK rats). Molecular weights of the respective proteins are shown in kilo Dalton (kDa). Original western blot figures are shown in Figure S4, quantitation values shown in Table S4.

We further checked the effect of aspirin on the expression of cell-signaling regulatory protein, Akt in GK rats. As shown in Figure 8A, the phosphorylation activation of Akt was significantly inhibited in GK diabetic liver compared with control rat liver, and aspirin treatment enhanced the activation of this marker protein involved in insulin signaling. However, Akt activation, remained lower in the kidney of GK rats even after aspirin treatment. Aspirin treatment reduced Akt activation even in the control rats in the kidney suggesting a tissue-specific response in insulin signaling. Glut-4 plays an important role in glucose homeostasis and insulin sensitization. We observed decreased expression of Glut-4 in both the liver and kidney tissues (Figure 8B). Aspirin treatment significantly improved the expression in both the tissues suggesting improved insulin sensitivity. We also observed decreased phosphorylation of JNK in GK diabetic liver compared with control rat liver and no significant improvement after aspirin treatment (Figure 9). However, unlike the liver, no significant change in expression of JNK was observed in the kidney of GK rats, though a significant inhibition was observed after aspirin treatment. This suggests a tissue-specific differential activation/response in GK diabetic rats.

### 3.8. Effect of Aspirin on Intracellular Structural Integrity

**Figure 10:**
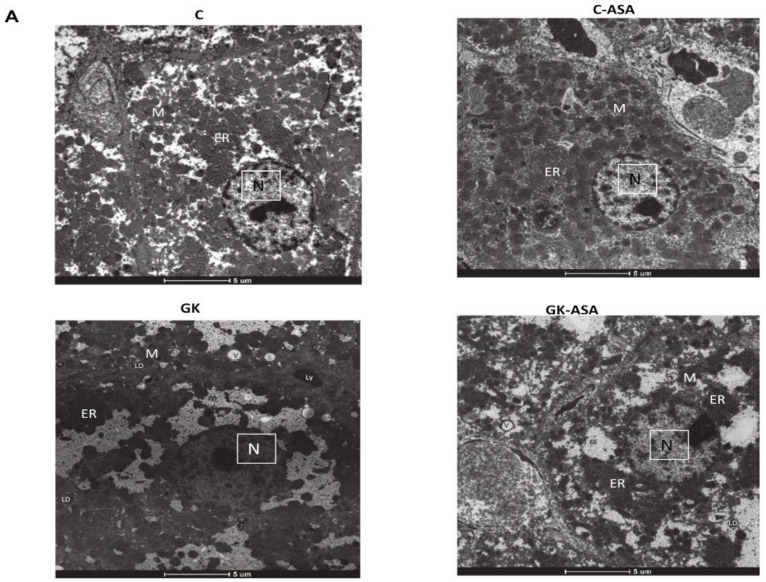

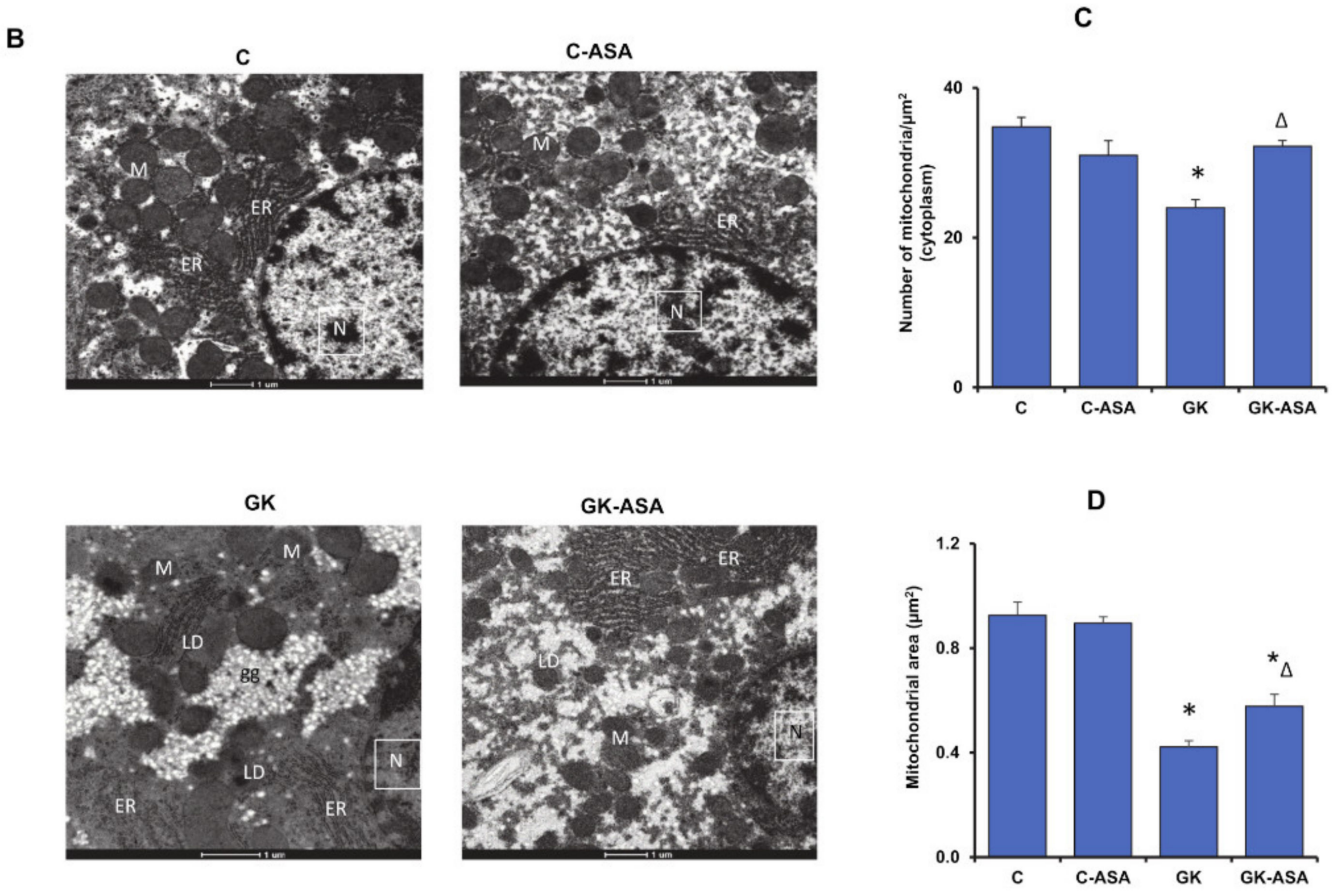
Effect of aspirin (ASA) on intracellular integrity of liver using electron microscopy. Liver samples from ASA-treated and untreated control and GK rats were fixed in Karnovsky’s fixative for ultrastructural imaging and processed as described in Materials and Methods. (**A**) shows the images under low magnification (scale bar = 5 μm) and (**B**) shows images under high magnification (scale bar = 1 μm). Note the large, coalescing glycogen granules are reduced after ASA administration. Moreover, ASA-treated GK rats showed numerous non-hypertrophied mitochondria compared with those in untreated in GK rats. N—nucleus, M—mitochondria, ER—endoplasmic reticulum, LD—lipid droplets, Ly—lysosomes, V—vacuoles, gg—glycogen granules. Figures are representative of at least 10 electron micrographs from each group. Histograms represent the number of mitochondria per µm^2^ of cytoplasm (**C**) and mitochondrial area (µm^2^) (**D**). Quantitation was done using ImageJ 1.53e software (National Institute of Health). At least 10 electron micrographs were analyzed for each group and the mean calculated. Asterisks indicate significant difference, which was fixed as *p* < 0.05 (* indicates *p* < 0.05 compared with control and Δ indicates *p* < 0.05 compared with GK rats).

**Figure 11:**
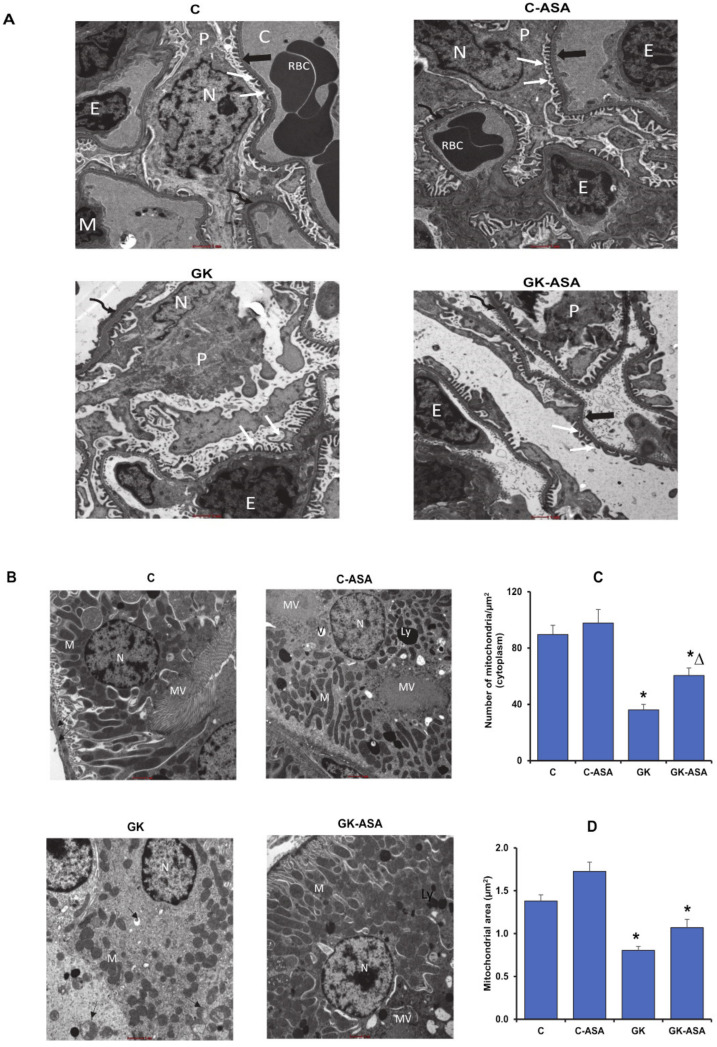
Effect of aspirin (ASA) on intracellular integrity of kidney using electron microscopy. Electron microscopy images of kidney demonstrating the renal glomerular corpuscle (**A**) and the proximal tubules (**B**) of control and GK rats treated with/without aspirin are shown. Tissue samples from kidney of ASA-treated and untreated control and GK rats were fixed in Karnovsky’s fixative for ultrastructural imaging and processed as described in Materials and Methods. Note that the glomerular basement membrane (thin, curved arrow) is thinner in GK-treated rats compared with GK control rats (**A**). The number of mitochondria appears to be more numerous in ASA-treated rats when compared to their respective controls (**B**). M–mitochondria, MV–microvilli, V–vacuole, Ly–lysosome, P—podocytes, N—podocyte nucleus, E—endothelial cell, C—glomerular capillaries, foot processes (white arrows), fenestrated endothelium (dark bold arrow), basement membrane (thin, curved arrow). Scale bar = 1 μm (Figure 11A); 1 μm (**B**). Figures are representative of at least 10 electron micrographs from each group. Histograms represent the number of mitochondria per µm^2^ of cytoplasm (**C**) and mitochondrial area (µm^2^) (**D**). Quantitation was done using ImageJ 1.53e software (National Institute of Health). At least 10 electron micrographs were analyzed for each group and the mean calculated. Asterisks indicate significant difference, which was fixed as *p* < 0.05 (* indicates *p* < 0.05 compared with control and Δ indicates *p* < 0.05 compared with GK rats).

To check the intracellular integrity after aspirin treatment, electron microscopy of control and GK rat liver and kidney, with/without aspirin treatment, was performed (Figure 10 and Figure 11). GK rat liver showed a drastic accumulation of glycogen granules, a characteristic feature of T2D, compared with control rats. Nucleus appeared shrunken and indented with clumped chromatin (Figure 10A—low magnification) compared with the rounded euchromatic nucleus in the control rat liver. Cytoplasm appeared vacuolated and contained lysosomes and lipid droplets in GK rat liver. Some abnormalities in the integrity of intracellular organelles such as swelling and disintegration of mitochondria and fragmentation of rough endoplasmic reticulum (ER) were also observed in GK diabetic rat tissues (Figure 10B—high magnification) compared with the rounded or oval mitochondria and organized and conspicuous rough endoplasmic reticulum observed in the control rat liver. Aspirin treatment resulted in a reduction of glycogen granules accumulation. Morphological damage of mitochondria recovered after aspirin treatment, though some mitochondria still showed loss of cristae and a few lipid droplets were still present. In addition, normal nucleus and normal distribution of cisternae in the endoplasmic reticulum were also observed, suggesting beneficial effects of aspirin (Figure 10A,B). A significant decrease in mitochondrial number and mitochondrial area was seen in the liver of GK rats, which showed improvement after aspirin treatment (Figure 10C,D).

Electron microscopic examination of the renal glomerulus in GK rats showed hypertrophic and detached podocytes with shrunken nuclei. Irregular fusion of foot processes and unevenly fenestrated endothelium along with thickened basement membrane were also observed (Figure 11A). The proximal tubules showed degenerated and detached microvilli, mitochondria were swollen and vacuolated and nucleus showed condensed and unevenly distributed chromatin. Widely distended cytoplasmic spaces showed degenerated, broken organelles that projected into the lumen (Figure 11B). Aspirin treatment normalized the filtration barrier consisting of the podocytes and foot processes in the renal glomerulus. Regular fenestrated endothelium with uniformly thickened basement membrane were also observed (Figure 11A). The proximal convoluted tubules also showed regular, elongated, tightly packed mitochondria after aspirin treatment more than that observed in the control animals. A few lysosomes and vacuoles were still observed, which were also seen in the control rats treated with aspirin. ImageJ analysis showed significant reduction in the mitochondrial number and mitochondrial area in the kidney of GK rats and partial recovery was observed after aspirin treatment (Figure 11C,D).

## 4. Discussion

Metabolic reprograming and adaptation in energy metabolism are dynamic phenomena in the progression of diabetes. At the cellular and molecular levels, glucolipotoxicity in diabetes is associated with increased inflammation, oxidative stress and mitochondrial dysfunction, which make significant contributions in the development of metabolic complications/syndrome [22]. GK rats are an excellent model for studies on insulin-resistant diabetes owing to their milder hyperglycemia/hyperlipidemia and lack of severe obesity-related complications [2,32].

Aspirin and salicylates have been found to be beneficial in the GK rat model of diabetes [21,25,26,33]. The beneficial effects of ASA have been reported to be multifactorial and mediated by interfering with inflammation, oxidative stress, mitochondrial dysfunction, insulin secretion, insulin sensitivity and signaling and regulating energy metabolism in diabetes. A major concern with this group of drugs, however, is the frequency and severity of their gastrointestinal side-effects, which could be due to the inhibition of COX-1 [17]. However, studies using intraperitoneal injections of aspirin (100 mg/kg) in rats showed that COX inhibition was not associated with gastrointestinal damage [17,18]. We therefore used this dose and mode of injection in our study.

We have previously reported that intraperitoneal aspirin treatment with 100 mg/kg body weight for 5 weeks decreased inflammatory responses, insulinemia and hyperlipidemia and improved pancreatic β-cell function and glucose tolerance without any significant organ toxicity [21]. Our recent study [23] also showed aspirin-induced protection of the heart and pancreas of GK rats from oxidative and mitochondrial stress and redox imbalance. Our present study on the same cohort of animals demonstrated that aspirin treatment protects the liver and kidneys of these diabetic animals from alterations in redox homeostasis, oxidative stress, energy metabolism, mitochondrial morphology and function and insulin signaling.

Reactive oxygen species (ROS) produced in all eukaryotic cells regulate several physiological processes but when available in excess can react with lipids, proteins and nucleic acids causing extensive tissue dysfunction and injury [34]. Increased ROS production has been shown to trigger mitochondrial dysfunction, resulting in an amplified ROS signal [35]. In addition to the ROS produced in the affected mitochondria, ROS trafficking between mitochondria resulted in an elevated production of ROS, causing oxidative stress-induced injuries, thus promoting organ dysfunction [35,36]. Our results also showed increased NOX activity accompanied by increased LPO and a decrease in SOD activity in the liver and kidney of GK rats, suggesting increased oxidative stress. Cytoplasmic vacuolation has been attributed to lipid peroxidation due to oxidative stress that damages cell membrane and cell organelles [37]. Studies have also suggested lipid peroxidation as the cause of dilated endoplasmic reticulum in the liver of rats with NAFLD [38]. Firneisz [39] showed that NAFLD is closely related to and is the leading cause of chronic liver disease predicted by T2D, which has been shown to cause disruption in endoplasmic reticulum homeostasis (ER stress), which leads to mitochondrial dysfunction, oxidative stress, inflammation and cell death [40]. Researchers have also shown morphological complications in the kidney characterized by widening of Bowman’s spaces and thickening of basement membrane in the renal cortices and loss of apical microvilli with swollen mitochondria in the proximal convoluted tubules of streptozotocin-induced diabetic rats [41]. Our ultrastructural studies confirmed damage to the cell and organelles, including shrunken nucleus, vacuolated cytoplasm, fragmented rough endoplasmic reticulum (ER) and swelling and disintegration of mitochondria (as seen by the decrease in mitochondrial number and area) in the liver of GK rats. In addition, kidney of these animals showed unevenly fenestrated endothelium with thickened basement membrane in the renal glomerulus and degenerated and detached microvilli and swollen and vacuolated mitochondria, as well as decreased mitochondrial number and area in the proximal tubules. These abnormalities could be due to the increased oxidative stress in the diabetic animals. Aspirin treatment helped to normalize these changes. The normal distribution of cisternae and organized endoplasmic reticulum in the GK rat liver after aspirin treatment suggests significant improvement in cellular functions. This is consistent with earlier studies which showed that increased granules on the rough endoplasmic reticulum suggested improved cell function [42]. The intracellular organelles in the kidney also showed marked morphological improvement in the glomerulus as well as the proximal tubules after aspirin treatment. Lysosomes are known to phagocytose degraded and damaged organelles to manage cellular homeostasis under oxidative and endoplasmic stress [43]. This could explain the increased number of lysosomes observed in the proximal convoluted tubules of aspirin-treated GK rats.

GSH-dependent redox metabolism was also altered in GK rat tissues as demonstrated by the reduced level of GSH and its metabolizing enzymes, GST and GSH-Px and increase in the activity of GSH-reductase. Aspirin treatment restored most of these changes, suggesting activation of the antioxidant redox metabolism. Similarly, the depleted expression of HO-1, an antioxidant responsive protein, was also increased by aspirin treatment, in both the liver and kidney of GK rats. These results are consistent with previous studies showing enhanced antioxidative defense mechanisms in diabetic rats treated with aspirin [44,45,46]. In addition, our studies on selected CYP 450s, CYP 2E1 and CYP 3A4 have demonstrated that these enzyme activities were markedly increased in diabetes, confirming our earlier reports on the changes in CYP 450s [8,10,11]. Aspirin treatment reduced enzyme activity close to normal values, suggesting that aspirin has beneficial effects on restoring the altered metabolism of endogenous and exogenous substrates, which were adversely affected in GK diabetic rats.

Aspirin treatment appeared to reprogram the altered glucose metabolism in the cytosol as well as in the mitochondria. Our results showed an increase in cytosolic hexokinase activity in GK rat liver and kidney, which was reduced after aspirin treatment. Aspirin treatment also increased enzyme activity in control non-diabetic rat tissues, suggesting an increased utilization of glucose, which might have relevance to the hypoglycemic action of aspirin. The increased hepatic glucose utilization was also supported by our electron microscopic studies, which showed a reduction in the hepatic glycogen pool after aspirin treatment. These results are in agreement with a previous study on glycogen depletion in diabetic rat tissues after aspirin treatment [47]. Researchers have demonstrated increased lipid droplets in the liver of rats administered glucose or high-fat diets [48,49]. Our results also showed increased lipid droplets in the GK diabetic rat liver, which were decreased after aspirin treatment.

Phosphorylation of Akt substrate leads to the translocation of Glut-4 to the plasma membrane, promoting extracellular glucose uptake resulting in decreased blood glucose and improving insulin resistance-induced diabetic symptoms [50,51]. Our study also showed a moderate activation of depleted Akt and Glut-4 expression in the liver, suggesting increased insulin sensitivity and glucose uptake after aspirin treatment. However, aspirin treatment showed a less significant effect on the reduced expression of Akt in the kidney, suggesting tissue-specific responses in insulin signaling and energy metabolism. A decreased expression of p-Akt was also observed in the control non-diabetic rat tissues treated with aspirin. This is consistent with the results of a previous study showing decreased expression of Akt, along with ERK and PI3K, after treatment with different doses of aspirin, in a rat model of acute pulmonary embolism, suggesting that downregulation of these signaling pathways may, in turn, inhibit the expression of inflammatory cytokines [52]. However, an increase in Glut-4 expression was observed in the kidney after aspirin treatment, suggesting independent regulation of Glut-4 in the kidney. On the other hand, reduced expression of p-JNK, a serine kinase, was observed in GK rat liver but not in the kidney. Aspirin treatment partially reversed this affect, again suggesting differential insulin signaling/energy metabolism responses in these tissues. Researchers have also shown that PI3/Akt phosphorylation is down regulated in diabetes [53]. The inhibition of JNK improves Akt phosphorylation, which in turn, downregulates glycogen synthase kinase phosphorylation [54]. Studies have also shown that oxidative stress-induced factors like hydrogen peroxide and byproducts such as JNK are activated by oxidative damage and can suppress Glut-4 expression [55,56,57].

Day [58] hypothesized that the release of ROS accelerates hepatocellular injury by decreasing the activities of the mitochondrial respiratory chain enzymes and the Krebs’ cycle enzyme, glutamate dehydrogenase (GDH) (glutamate to α-ketoglutarate). In our study, we observed that GDH was inhibited in the liver and kidney of GK rats. Aspirin treatment partially restored the enzyme activity. GDH is known to be allosterically regulated by diverse compounds, including purine nucleotides [59]. Thus, under conditions of high energy levels, GDH is inhibited, whereas energy depletion activates GDH, providing α-ketoglutarate, for the citric acid cycle [60]. Studies have suggested that protection of GDH increases insulin signaling and mitochondrial function, thus preventing diabetic-induced toxicities in the tissues [61]. Hyperglycemia has been shown to cause alterations and dysfunction in the mitochondria and ultra-structural abnormalities in the mitochondria as well as other organelles [62,63,64,65]. We also observed swelling and disintegration of mitochondria and shrunken, indented nucleus with clumped chromatin in the GK diabetic rat liver and kidney. This could be attributed to mitochondrial dysfunction caused by decreased oxidative phosphorylation leading to decreased ATP production [34]. Our results demonstrate improvement in the morphology of the nucleus and moderate increase in the number and area of mitochondria, which, in turn, helped activate the mitochondrial respiratory enzyme complexes I, II/III and IV, resulting in increased ATP synthesis after aspirin treatment in the liver and kidney. Studies have shown that aspirin can promote mitochondrial biogenesis, which, in turn, profoundly affects mitochondrial metabolism and energy utilization [66,67]. This supports our findings of increase in mitochondrial number and ATP synthesis after aspirin treatment. Researchers have shown that damaged mitochondria and other organelles are removed from the cell by autophagy (mitophagy) to prevent the release of pro-apoptotic proteins from the mitochondria and prevent ATP depletion [68]. This again supports our results of increased lysosomes observed in the proximal convoluted tubules of aspirin-treated GK rats. A balance between mitochondrial biogenesis and mitophagy helps in maintaining a constant number of functionally active mitochondria in the cell [64].

## 5. Conclusions

Increased glucose metabolism and activation of insulin signaling markers were observed in GK diabetic rats treated with aspirin. Aspirin appears to exert multifactorial beneficial effects by reprogramming redox and energy metabolism and partially restoring mitochondrial bioenergetics. Aspirin also had a positive influence on CYP 450 activity in diabetic rats, which exhibited altered metabolism of xenobiotic and endogenous substrates. However, a balanced study in terms of the benefit/risk ratio is required before treatment with aspirin alone or in combination with other antidiabetic drugs, in the therapeutic management of diabetes.

## 6. Limitations

In our present study, we have shown that aspirin improved redox and energy metabolism and restored mitochondrial bioenergetics in GK diabetic rats. However, a detailed pharmacokinetic study is required to compare the metabolism of aspirin in normal Wistar and GK diabetic rats to correlate the efficacy/toxicity of this drug.

## Data Availability

The data used to support the findings of this study are included in the manuscript.

## Supplementary Materials

The following supporting information can be downloaded at: https://www.mdpi.com/article/10.3390/life12010104/s1, Figure S1. Original Western blot images for Figure 7. Figure S2. Original Western blot images for Figure 8A. Figure S3. Original Western blot images for Figure 8B. Figure S4. Original Western blot images for Figure 9. Table S1. Quantitation values for Western blots for Figure 7. Table S2. Quantitation values for Western blots for Figure 8A. Table S3. Quantitation values for Western blots for Figure 8B. Table S4. Quantitation values for Western blots for Figure 9.
