# Supplementary material for: Alterations in Energy Metabolism, Mitochondrial Function and Redox Homeostasis in GK Diabetic Rat Tissues Treated with Aspirin
Source: Life (Basel). 2022 Jan 12;12(1):104. doi: 10.3390/life12010104 (PMC8780217; doi:10.3390/life12010104)
Supplement: Supplementary file 1 [file life-12-00104-s001.zip › supplementary material.pdf]

Supplementary materials

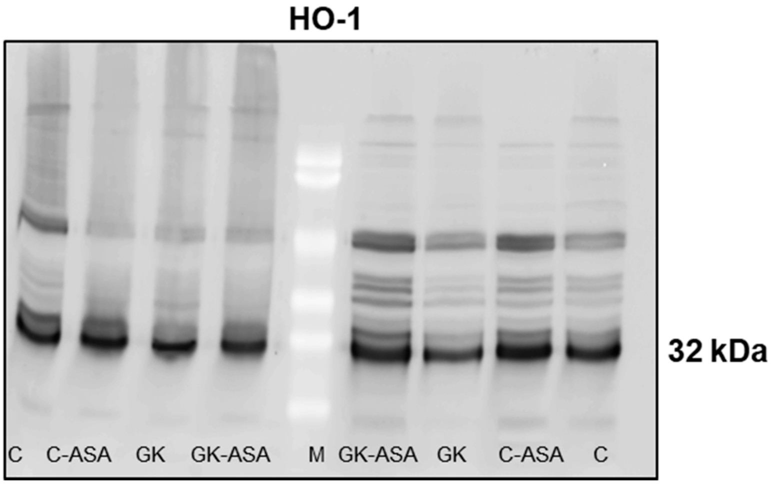

(**a**)

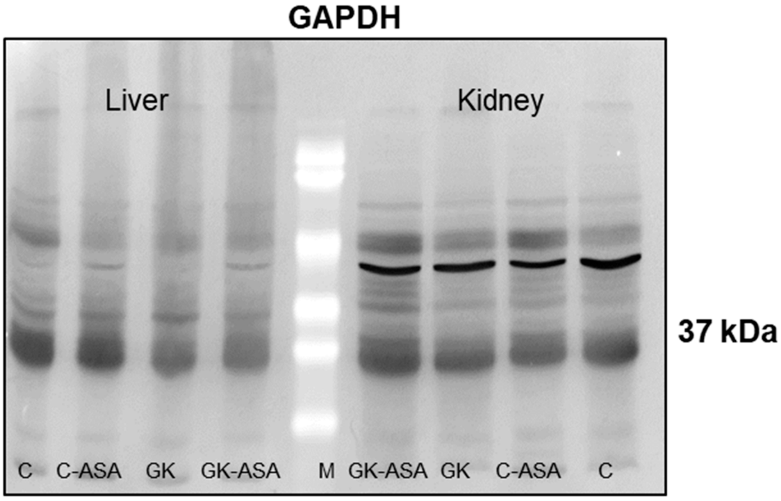

(**b**)

Figure S1. The blots were developed using ECL Plus Western Blotting Luminol Reagent kit and the bands visualized using the Typhoon FLA 9500 system (GE Healthcare, Uppsala, Sweden). Fig 7 was generated from these blot images. M-molecular weight marker.

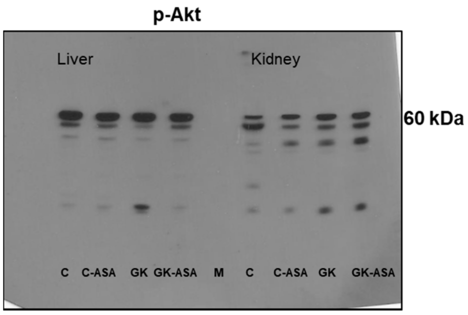

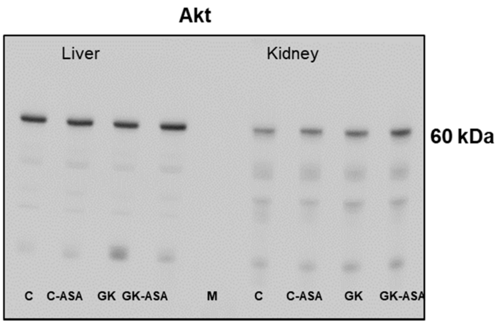

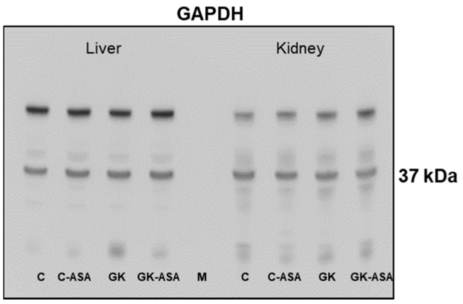

Figure S2. The blots were developed using ECL Plus Western Blotting Luminol Reagent kit and the bands visualized using X-ray films or Typhoon FLA 9500 system (GE Healthcare, Uppsala, Sweden). Figure 8A was generated from these blot images. M-molecular weight marker.

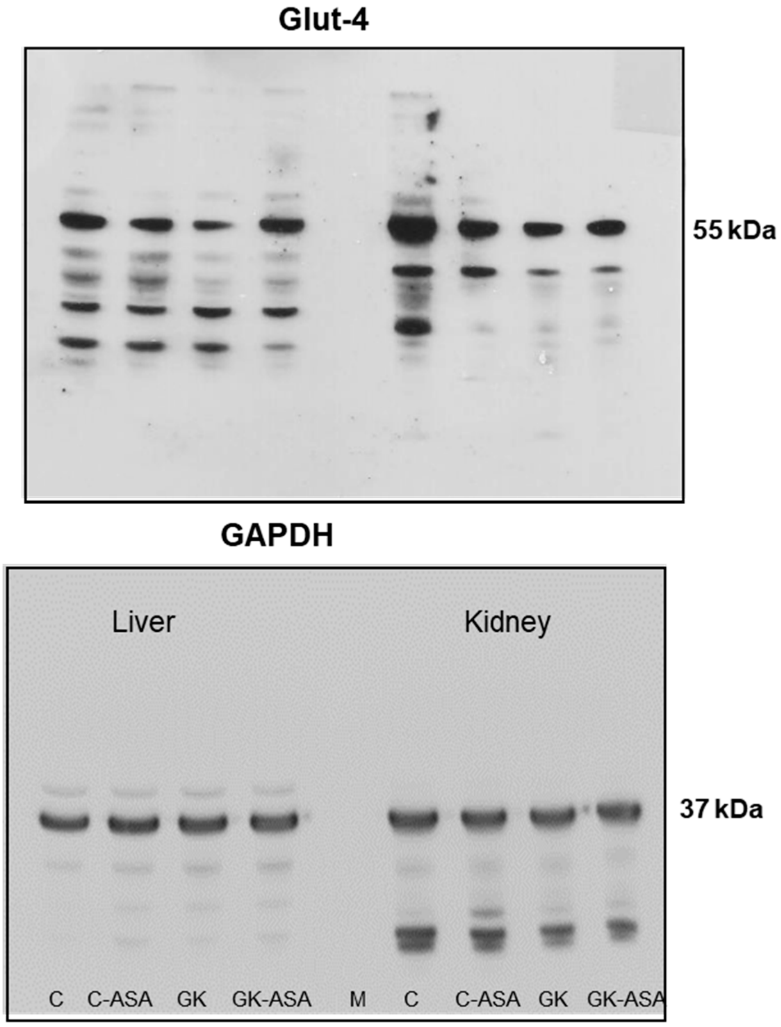

Figure S3. The blots were developed using ECL Plus Western Blotting Luminol Reagent kit and the bands visualized using X-ray films or Typhoon FLA 9500 system (GE Healthcare, Uppsala, Sweden). Figure 8B was generated from these blot images. M-molecular weight marker.

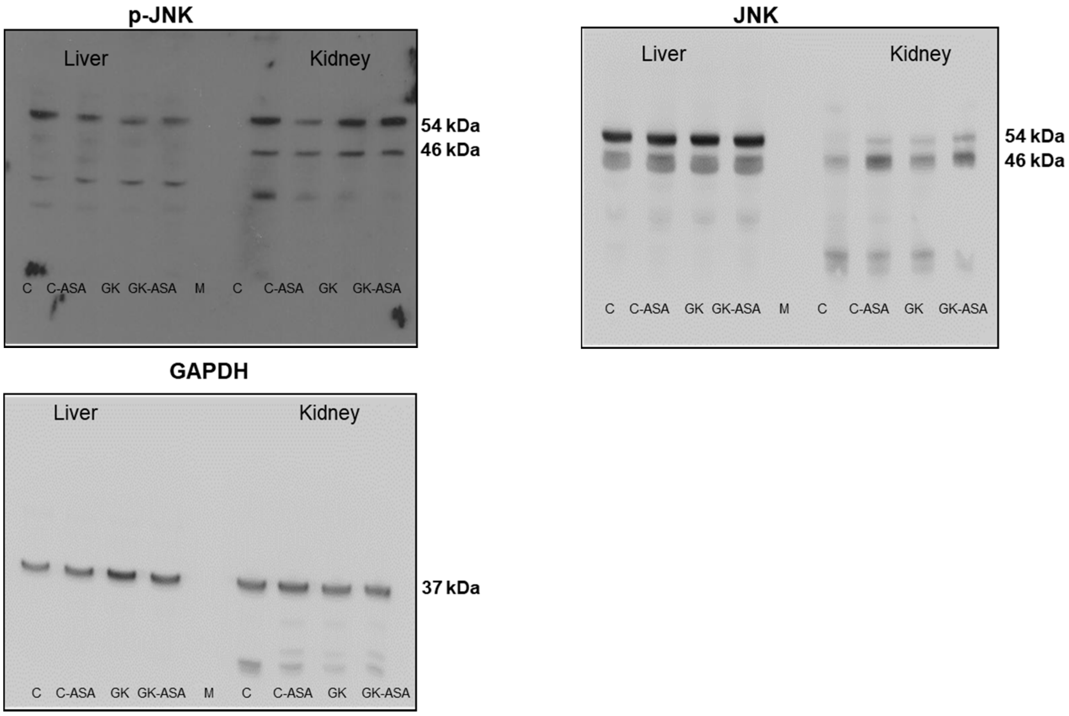

Figure S4. The blots were developed using ECL Plus Western Blotting Luminol Reagent kit and the bands visualized using X-ray films or Typhoon FLA 9500 system (GE Healthcare, Uppsala, Sweden). Figure 9 was generated from these blot images. . M-molecular weight.
